# The Impact of Anthropic and Natural Events on Leishmaniasis Burden, Control Measures, and Public Health Importance

**DOI:** 10.1155/tbed/7588132

**Published:** 2025-09-18

**Authors:** Ahmad Khosravi, Iraj Sharifi, Mehdi Bamorovat, Maryam Hakimi Parizi, Mohammad Reza Aflatoonian, Fatemeh Sharifi, Setareh Agha Kuchak Afshari, Abbas Aghaei Afshar, Baharak Akhtardanesh, Elahe Mollaakbari, Mahsa Faramarzpour, Mohammad Reza Shirzadi, Ali Khamesipour, Mehdi Mohebali

**Affiliations:** ^1^Leishmaniasis Research Center, Kerman University of Medical Sciences, Kerman, Iran; ^2^Research Center of Tropical and Infectious Diseases, Kerman University of Medical Sciences, Kerman, Iran; ^3^Medical Mycology and Bacteriology Research Center, Kerman University of Medical Sciences, Kerman, Iran; ^4^Department of Clinical Sciences, School of Veterinary Medicine, Shahid Bahonar University of Kerman, Kerman, Iran; ^5^Center for Communicable Diseases Control, Ministry of Health and Medical Education, Tehran, Iran; ^6^Center for Research and Training in Skin Diseases and Leprosy, Tehran University of Medical Sciences, Tehran, Iran; ^7^Department of Medical Parasitology and Mycology, School of Public Health, Tehran University of Medical Sciences, Tehran, Iran

**Keywords:** control measures, disasters, environmental changes, leishmaniasis, public health importance

## Abstract

Leishmaniasis is an important neglected disease among infectious diseases, which is highly affected by diverse adverse conditions. Due to the intricate nature of *Leishmania*, the impact of the disease remains significant. In this review, we aim to assess the role of these factors that influence disease trends and introduce preventive and control strategies, highlighting their importance in public health issues. Numerous influencing factors, such as trade and travel, civil unrest, migration, low socioeconomic status, peri-urban settlements, ecological changes, climate variations, and many other confounding factors, play significant roles in the transmission and distribution of leishmaniasis. On the other hand, inadequate infrastructure and disruptions in the health system services are closely linked with the incidence of leishmaniasis in vulnerable populations. These circumstances contribute to the alteration of vector and reservoir compositions, land degradation, agricultural setbacks, water scarcity, and livestock depletion, resulting in increased infectious and noninfectious disease conditions. In conclusion, significant anthropic and natural environmental changes have the potential to trigger leishmaniasis outbreaks in established endemic areas and lead to the emergence/reemergence of new foci in previously unaffected regions. Consequently, enhancing public health awareness, particularly in high-risk regions, along with implementing active and passive surveillance, ensuring early diagnosis, and providing timely and effective treatment for patients, are vital measures for controlling the disease.

## 1. Background

Leishmaniasis, a neglected tropical disease caused by protozoan parasites of the *Leishmania* genus, poses a significant public health challenge globally [1]. This vector-borne disease affects millions of people in endemic areas, with varying clinical forms ranging from cutaneous (CL) to visceral (VL) manifestations. CL causes skin sores, mucocutaneous (MCL) lesions that affect the mucous membranes of the nose and throat, presenting a mutilating form, while VL is a systemic disease that affects reticuloendothelial organs, and over 95% can be fatal if left untreated [2]. The disease not only leads to morbidity and mortality but also carries social and economic burdens in endemic areas [3]. Addressing leishmaniasis through effective prevention, diagnosis, and treatment strategies is essential to mitigate its public health implications and improve the well-being of affected communities.

The global burden of leishmaniasis is estimated to be around 1.3 million new cases per year, with approximately 30,000 deaths annually. The disease is endemic in more than 98 countries, predominantly in tropical and subtropical regions of Asia, Latin America, Africa, and Europe [4]. Control and prevention efforts are crucial in reducing the incidence and impact of leishmaniasis. This includes vector control measures, early diagnosis, effective treatment, and community education. Research continues to focus on developing new diagnostic tools, treatment options, and potential vaccines to combat this neglected tropical disease [5]. Together, these measures seek to break the transmission cycle and alleviate the impact of leishmaniasis through a coordinated, forward-looking strategy involving multiple sectors.

Several challenges exist in controlling leishmaniasis [6], including (i) Limited resources: Many regions affected by leishmaniasis have limited healthcare infrastructure, funding, and resources for disease control programs. (ii) Environmental management for vector control: Controlling the sandfly vector can be challenging due to its widespread distribution, adaptability, and resistance to insecticides. (iii) Diagnostic limitations: Diagnosing leishmaniasis accurately can be difficult, especially in areas with limited access to laboratory facilities and trained healthcare professionals. (iv) Limited treatment options: Some forms of leishmaniasis require long and costly treatment regimens with drugs that may have toxic adverse effects, and drug resistance is also a concern. (v) Lack of effective vaccines: Currently, there are no licensed vaccines available for leishmaniasis, making prevention solely dependent on vector control measures. (vi) Socioeconomic factors: Poverty, malnutrition, lack of sanitation, deficient selective waste collection, and displacement due to conflicts can contribute to the increased risk of leishmaniasis transmission [7]. In addition, the great diversity of parasites, vectors, reservoirs, and ecological and epidemiological scenarios involved in the transmission of leishmaniasis precludes generalizations and requires strategies adapted to each local situation

Addressing these challenges requires a multifaceted approach that involves collaboration between governments, healthcare providers, researchers, and international organizations to improve surveillance, diagnosis, treatment, and prevention strategies for leishmaniasis.

The economic impact of leishmaniasis is substantial, as it often affects individuals in their productive years, leading to disability and loss of income. The costs associated with diagnosis, treatment, and management can also place a financial strain on healthcare systems and affected households. Strengthening surveillance systems and healthcare infrastructure is essential to effectively manage the disease and reduce its impact on public health Collaboration between governments, healthcare organizations, research institutions, and nongovernmental organizations is key to addressing the challenges posed by this neglected tropical disease [8].

Control strategies against diverse vectors and so many reservoirs have been impractical. The Global Vector Control Response 2017–2030 (GVCR) introduces a novel approach to enhance vector control globally by boosting capacity, enhancing surveillance, improving coordination, and fostering integrated actions across various sectors and diseases [9]. Partial success has been obtained in some countries, like Brazil [10]. Despite numerous adverse effects, chemotherapy has been the mainstay of leishmaniasis management approaches [11]. In the present review, we intend to evaluate the effects of natural and man-made events on the occurrence and spread of leishmaniasis. The goal is to pinpoint critical factors that shape disease patterns and to guide the development of preventive and control measures and their public health significance.

## 2. Search Strategy

We searched for the impact of natural and man-made circumstances on leishmaniasis and considered using the following search strategies. We used keywords like “leishmaniasis,” “natural disasters,” “man-made events,” “impact,” “epidemiology,” and specific event names (e.g., floods, earthquakes, deforestation). Furthermore, we used scientific databases like PubMed, Scopus, Web of Science, and research articles on the topic. We also looked for review articles or meta-analyses that summarized existing research on the impact of naturally occurring and artificial incidents on leishmaniasis.

## 3. Definition of Terms

Anthropic events: Encompass changes or occurrences directly resulting from human activities. These were further grouped by the nature of the activity or its primary consequence (e.g., urbanization leading to environmental and social changes, migration due to conflict or economic factors, agricultural projects altering land use). The logic here was to identify how specific human actions create pathways or conditions that can amplify leishmaniasis transmission.

Natural events: On the other hand, are those driven by natural processes, such as climate change or geological activity. The analytical focus here was on how these events directly or indirectly modify environmental conditions, potentially affecting vector habitats, reservoir host distribution, and human vulnerability.

Unplanned urbanization: The factor is the rapid and unmanaged growth. The impact on leishmaniasis risk can be seen through increased population density (potentially quantifiable), poor sanitation (qualitative but with proxies like access to sewage systems), and altered human–vector interaction (challenging to quantify directly but inferable from incidence data in such areas).

Climate change: Includes changes in temperature and precipitation (quantifiable). The impact on leishmaniasis could be a shift in the geographic range of sandfly vectors (potentially modeled and quantified) or changes in the duration of the transmission season.

Preventive and control strategies: We aimed to highlight how understanding these influencing factors can inform targeted interventions. For example, recognizing unplanned urbanization as a risk factor suggests the need for urban planning that incorporates vector control and improved sanitation. Similarly, understanding the impact of migration necessitates surveillance and healthcare provision for displaced populations.

Risk factors: The risk of leishmaniasis infection denotes the likelihood of individuals or populations contracting *Leishmania* infection, primarily transmitted through the bite of infected female phlebotomine sandflies. This risk is shaped by a complex interplay of factors, including geographic distribution, environmental and climatic conditions, vector prevalence and behavior, patterns of human exposure, socioeconomic conditions, host immune status, and the presence of reservoir hosts.

Impact of leishmaniasis affliction: The impact of leishmaniasis affliction encompasses the wide-ranging health, social, and economic consequences resulting from infection with *Leishmania* parasites. Clinically, the disease manifests in CL, MCL, or VL forms, each associated with varying degrees of morbidity, disfigurement, and, in the case of VL leishmaniasis, significant mortality if left untreated. The physical effects often lead to chronic pain, secondary infections, and long-term scarring, particularly in CL forms. Beyond clinical outcomes, leishmaniasis exerts a profound socioeconomic burden, particularly in endemic regions characterized by poverty and limited healthcare access. The disease often leads to reduced productivity, social stigma, loss of income, and increased healthcare expenditures for affected individuals and communities. In children and immunocompromised individuals, the infection can be especially severe, contributing to malnutrition, developmental delays, and increased vulnerability to other diseases.

## 4. Life Cycle

**Figure 1:**
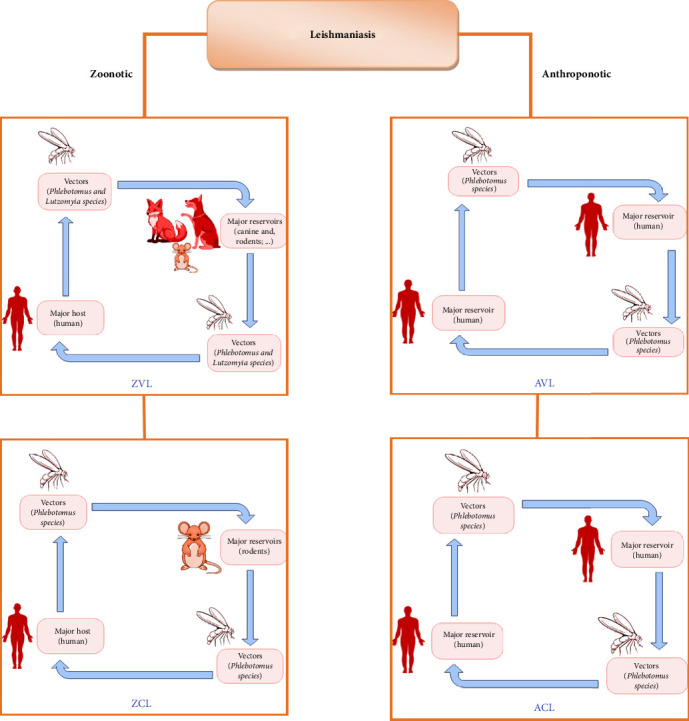
Illustrating four different types of *Leishmania* species life cycles. Zoonotic visceral leishmaniasis (ZVL), anthroponotic visceral leishmaniasis (AVL), zoonotic cutaneous leishmaniasis (ZCL), and anthroponotic cutaneous leishmaniasis (ACL).

The disease is spread when an infected female sandfly bites a mammalian reservoir, which usually includes rodents, sloths, marsupials, or wild or domestic canines (Figure 1) [12].

Different reservoir hosts and vectors of sandflies are involved in the spread and transmission of *Leishmania* spp. in different regions of the world, depending on the parasite and the epidemiological setting.

### 4.1. Vectors

Female phlebotomine sandfly species belonging to two genera transmit leishmaniasis: *Phlebotomus* in the Old World and *Lutzomyia* in the New World [13]. Over 90 sandfly species are established or probable vectors that are implicated in the life cycle. Sandflies are fairly poor fliers, simply distressed by wind, and live near animal reservoir hosts [14, 15]. Their biology includes a peak in crepuscular, short-hop flights, and extensive dispersion, which can reach a radius of up to 243 m [16].

### 4.2. Reservoir Hosts

**Figure 2:**
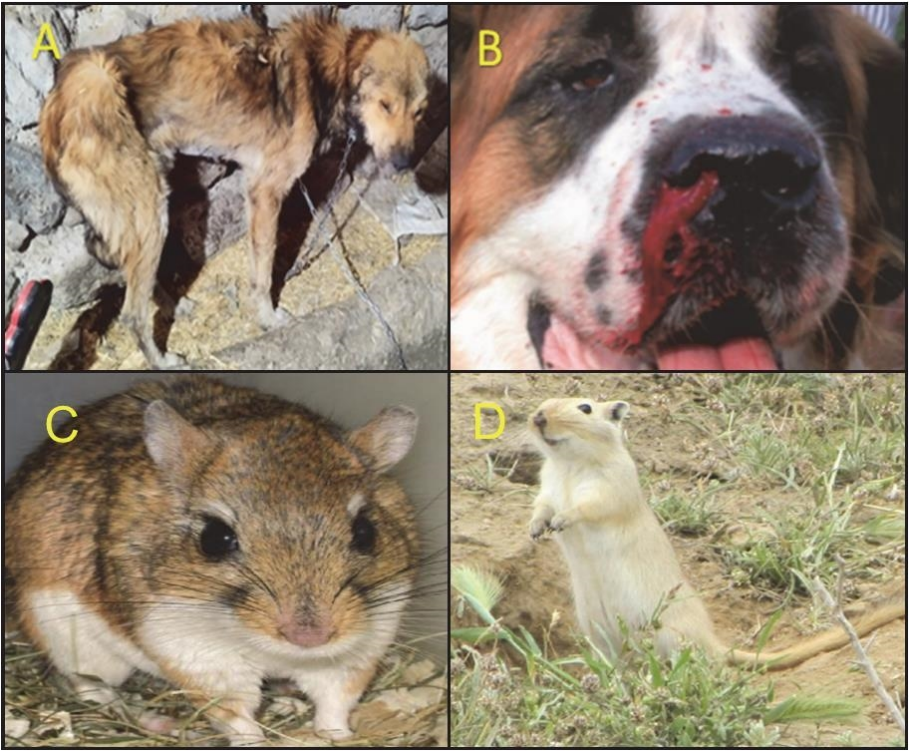
(A,B) Demonstrate main reservoir hosts of visceral leishmaniasis (canines, particularly dogs) presenting clinical symptoms and signs (severe emaciation, skin and eye lesions, poor appetite, enlargement of the spleen, liver, and lymph nodes, pancytopenia, weakness, excessive nail growth, and lameness). (C,D) Primary reservoir hosts (gerbils) of zoonotic cutaneous leishmaniasis display lesions on the naked parts of the body [17].

Leishmaniasis can be grouped into two broad categories according to the source of human infection: zoonotic leishmaniases, in which the reservoir hosts are domestic or sylvatic animals (Figure 2), and anthroponotic leishmaniases, in which the reservoir host is human. Although each *Leishmania* species normally falls into one or the other of these categories, occasional exceptions are seen. For example, CL caused by *L. donovani* and *L. tropica* is usually anthroponotic, but in some foci, it derives not from humans but from other animals. For several CL species that are typically zoonotic, humans may constitute an incidental source of infection [18, 19].

## 5. Natural and Anthropic Events

### 5.1. Anthropic Events

#### 5.1.1. Unplanned Urbanization

Unplanned urbanization can increase the risk of leishmaniasis due to various factors. Rapid and unplanned urban growth can lead to overcrowding, poor sanitation, inadequate housing conditions, and limited access to healthcare services, all of which create favorable environments for the spread of leishmaniasis. Additionally, urbanization can disrupt natural habitats and ecosystems, leading to increased contact between humans, reservoir hosts, and sandflies, which are vectors for the parasite that causes leishmaniasis. These factors combined can contribute to the higher incidence and prevalence of leishmaniasis in urban areas experiencing unplanned urbanization [6, 20].

To decrease the risk of unplanned urbanization, it is essential to implement effective urban planning strategies and policies. Some key approaches to mitigate this risk include: (i) Comprehensive urban planning: Develop and implement comprehensive urban planning frameworks that consider factors, such as population growth, infrastructure development, land use, and environmental sustainability. (ii) Zoning regulations: Enforce zoning regulations to guide the development of different areas within a city, ensuring that land is used appropriately and following the city's long-term goals. (iii) Infrastructure development: Invest in infrastructure development to support urban growth, including transportation systems, water supply, sanitation, and waste management facilities. (iv) Public participation: Involve local communities and stakeholders in the urban planning process to ensure that their needs and concerns are taken into account. (v) Land use management: Implement land use management strategies to control the expansion of urban areas and protect valuable natural resources [21, 22].

The lack of a proper waste disposal system can contribute to the spread of *Leishmania* parasites and increase the risk of leishmaniasis transmission. Improper waste management practices, such as open dumping of garbage or inadequate sanitation facilities, can create breeding grounds for sandflies, which are the vectors responsible for transmitting *Leishmania* species [23–26]. These sandflies thrive in environments with organic waste, humid and dark habitats, increasing the likelihood of human–vector contact and subsequent transmission of the parasite. Therefore, addressing issues related to waste disposal and sanitation is crucial in preventing the spread of leishmaniasis and reducing the burden of the disease in affected communities.

The role of civil debris and waste in exacerbating leishmaniasis is significant. Improper disposal of waste can create breeding grounds for sandflies, which are the vectors responsible for transmitting the *Leishmania* parasite [23, 27–30]. To control the risk of civil debris and waste contributing to the spread of leishmaniasis, several measures can be implemented: (i) Implement proper waste management practices: Ensure regular collection and disposal of waste to prevent the accumulation of debris that can serve as breeding grounds for sandflies. (ii) Promote community awareness: Educate the community about the importance of proper waste disposal and sanitation practices to reduce the risk of leishmaniasis transmission. (iii) Use insecticides: Apply insecticides to areas where sandflies are likely to breed, such as in and around waste disposal sites, to control the vector population. (iv) Improve sanitation infrastructure: Invest in infrastructure improvements, such as proper drainage systems and waste disposal facilities, to minimize the risk of exposure to leishmaniasis [31, 32].

#### 5.1.2. Peri-Urban Settlements

Peri-urban communities are places where urban and rural features converge on the periphery of urban centers. These regions frequently see fast population increase, the emergence of informal housing, and restricted access to infrastructure and essential services. Their closeness to natural ecosystems where sandflies flourish, poor waste management, decomposing organic matter, inadequate sanitation, crowded housing, a lack of clean water, and poor living conditions can all increase their risk of acquiring leishmaniasis. Additionally, peri-urban activities like farming, ranching, and deforestation might increase the likelihood of coming into contact with sandflies [6]. People who reside in peri-urban communities should take precautions, including applying insect repellent, donning protective clothes, and using impregnated bednets [33, 34].

To decrease the risk of contracting leishmaniasis in peri-urban settlements, several preventive measures can be implemented: (i) Vector control: Implementing vector control measures, such as insecticide spraying, use of bed nets, and environmental modifications to reduce resting and breeding sites for sandflies, which are the vectors transmitting the *Leishmania* parasite. (ii) Health education: Educating the households in the community about the transmission of leishmaniasis, symptoms, and preventive measures can help raise awareness and promote behavior changes to reduce the risk of infection. (iii) Improved housing and sanitation: Improving housing conditions, for instance, sealing cracks in walls and floors to prevent sandflies from entering homes, and ensuring proper sanitation practices can help reduce the risk of exposure to the parasite [35–37]. (iv) Community engagement: Engaging the community in participatory approaches to vector control and health promotion can help create sustainable interventions tailored to the specific needs of peri-urban settlements. (v) Early detection, prompt diagnosis, and effective treatment: Ensuring access to healthcare for leishmaniasis cases can help prevent the spread of the disease within the community [38, 39].

#### 5.1.3. Migration/Population Displacement

Leishmaniasis is one of the infectious illnesses that can spread due to migration, since people who move from endemic areas to metropolitan areas may transfer the parasite to new habitats. Urban locations might have factors, including overcrowding, substandard living conditions, and limited availability of public health care, that can worsen leishmaniasis transmission. It is true that leishmaniasis, a parasitic illness spread by sandfly bites, can become more likely to strike during migration. Individuals who move from non-endemic to endemic areas, or vice versa, have a higher risk of contracting the *Leishmania* parasite from various sandfly species [40, 41]. Addressing a variety of intricate social, political, and economic issues is necessary to reduce migration. Investing in economic development in areas with high migration rates, expanding access to employment and education opportunities, tackling the underlying causes of instability and violence, and enacting just and compassionate immigration laws are a few possible tactics. It is critical to take into account the particulars of each scenario and strive toward long-term solutions that put the welfare of people and communities first. Natural catastrophes or violence may also contribute to the leishmaniasis outbreak among migrating populations. Immigrants and medical professionals must understand the hazards and take precautions to lessen the disease's spread [42, 43].

According to the most recent data, a considerable number of people are annually displaced globally as a result of war and civil conflict [44]. This figure encompasses refugees, internally displaced persons, and asylum seekers who have been compelled to escape their homes because of violence, persecution, and conflict. The situation is dynamic and ever-changing, with ongoing conflicts and crises contributing to the displacement of millions of people worldwide [45, 46]. Addressing the causes of conflict, promoting peacebuilding initiatives, and offering assistance to displaced populations are essential components of resolving this humanitarian crisis. According to the most recent data, the number of people displaced worldwide as a result of drought is estimated to be tens of millions [24].

To address the issue of migration, it is essential to focus on creating sustainable and inclusive environments that provide opportunities and resources for residents to thrive in their communities. Some strategies to make people more likely to stay in their current location include: (i) Economic development: Promoting economic growth and job opportunities in the region can provide residents with stable employment and income, reducing the need to migrate in search of better prospects. (ii) Infrastructure development: Investing in infrastructure such as healthcare, education, transportation, and basic services can improve the quality of life for residents and make the area more attractive for long-term settlement. (iii) Social services: Providing access to social services, including healthcare, education, and social support programs, can enhance the well-being of residents and strengthen community ties. (iv) Community engagement: Encouraging community participation and involvement in decision-making processes can foster a sense of belonging and ownership among residents, leading to increased attachment to their locality. (v) Environmental sustainability: Promoting sustainable practices and environmental conservation efforts can help preserve natural resources and create a healthier and more resilient environment for residents to live in [7, 35, 47–49].

Movement across borders may increase the chance of infectious illnesses like leishmaniasis spreading. People who travel across borders may unintentionally bring the parasite or infected sandflies with them, spreading the illness to new areas where it may not have been before. This may cause leishmaniasis to spread to previously uninfected regions and give rise to new epidemics. Public health officials must keep an eye on and regulate people's and products' cross-border movement to stop the spread of infectious illnesses like leishmaniasis. Preventing the transmission of leishmaniasis involves implementing comprehensive public health measures, such as vector control, early diagnosis, treatment, and community education.

#### 5.1.4. Agricultural Projects, Construction, and Seasonal Workers

Agricultural projects, construction, and seasonal workers represent key factors in altering the dynamics of disease transmission. These activities often result in; Increased exposure to infectious agents: Workers are frequently in direct contact with soil, water, or animals that may harbor pathogens. Increased susceptibility: Particularly among seasonal workers, susceptibility may be heightened due to factors such as poor nutritional status, limited access to healthcare, or underlying health conditions (e.g., malnourishment). Environmental modifications: Land clearing, water storage, deforestation, and construction activities can disrupt natural ecosystems, thereby increasing the abundance of vectors (e.g., mosquitoes, rodents) or reservoirs (e.g., wild animals) of disease. Dispersion of vectors or reservoirs: Movement of construction materials, migration of workers, or disturbance of habitats can aid in the geographical spread of vectors and pathogens into new areas.

Irrigation projects and changes in land use can disrupt the natural ecosystem, leading to an increase in sandfly breeding sites and human–vector contact. Additionally, agricultural workers and individuals living close to agricultural areas are at higher risk of exposure to sandflies and, consequently, the *Leishmania* organisms. Agricultural workers may be exposed to sandflies during outdoor activities, such as planting, harvesting, and handling crops, especially.

To mitigate the risk of leishmaniasis through agricultural projects, it is important to implement preventive measures such as (i) Use of insecticide spraying, insect repellents, and protective clothing: Encourage agricultural workers to use insect repellents and wear long sleeves, pants, and hats to reduce exposure to sandflies. The use of insecticides must be monitored due to environmental and consequent public health issues. (ii) Environmental management. (ii) Implement measures to reduce sandfly breeding sites, such as proper waste disposal, vegetation management, and drainage of standing water. (iii) Health education: provide training and education to agricultural workers on the risks of leishmaniasis, symptoms to watch for, and preventive measures to reduce transmission. (iv) Collaboration between health authorities, agricultural agencies, and local communities is essential to comprehend these dynamics to implement effective prevention and control strategies to reduce the risk of leishmaniasis in agricultural settings [50, 51].

Construction workers can be at higher risk of contracting leishmaniasis through exposure to sandflies in endemic areas where the disease is prevalent [52, 53]. Factors, such as poor housing conditions, inadequate protection from sandflies, and lack of awareness about preventive measures, can contribute to the transmission and spread of leishmaniasis among these laborers [36, 54]. Implementing measures such as wearing protective clothing, using insect repellents, and improving housing conditions can help reduce the risk of leishmaniasis transmission in this occupational group [55].

#### 5.1.5. Trade and Transportation

The movement of diseased animals, such as dogs, rats, or wildlife, which can act as reservoir hosts for the parasite, is made possible by trade and transportation, which can also aid in the spread of leishmaniasis. Animals that are infected can be brought over international boundaries or to new places, spreading the illness to previously uninfected areas. Leishmaniasis can also spread geographically by the unintentional introduction of infected sandflies or their eggs into new ecosystems due to the worldwide traffic in animals and animal products. The spread of leishmaniasis through commerce and transportation can be reduced by tightening restrictions on animal movement and putting in place safeguards to detect and stop the entry of infected vectors [56–58].

#### 5.1.6. Cultural Habitats

A host's sleeping behaviors can indeed play a role in increasing the risk of leishmaniasis, particularly in regions where the disease is endemic and transmitted by sandflies. Additionally, sleeping outdoors or in poorly constructed shelters without adequate protection against sandflies, the vectors of leishmaniasis, can increase the likelihood of transmission. The domestic dog (*Canis familiaris*) is regarded as the primary urban reservoir for VL due to *L. infantum*, particularly in urban settings, as it carries a significant parasite load on its skin. This makes it a source of infection for sandfly vectors, thereby sustaining the disease [59].

To reduce the risk of leishmaniasis associated with sleeping behaviors, individuals can take the following preventive measures: (i) Sleep indoors in well-constructed housing with screens on windows and doors to prevent sandflies from entering. (ii) Use bed nets treated with insecticide and apply insect repellent before sleeping. (iii) Avoid sleeping outdoors, especially during peak sandfly activity times. (iv) Keep sleeping areas clean and free of debris that may attract sandflies. (v) Seek medical advice and take appropriate precautions before traveling to regions where leishmaniasis is endemic [55, 60, 61].

#### 5.1.7. Suboptimal Reporting

The underreporting of leishmaniasis cases poses challenges in accurately assessing disease burden, hindering effective surveillance and control efforts, and limiting targeted interventions [62, 63]. While suboptimal reporting is a widespread concern, resulting in an underestimated disease burden, a significant number of cases are still reported to the WHO [35]. WHO estimates that only 25%–45% of VL and 20%–50% of CL cases are officially reported, with many countries lacking formal reporting systems [64]. Addressing suboptimal reporting through enhanced surveillance systems, standardized case definitions, and increased awareness among healthcare providers is essential in tackling leishmaniasis.

#### 5.1.8. Misdiagnosis

**Figure 3:**
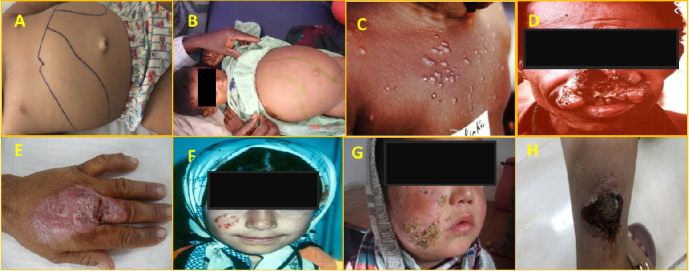
Representative images of visceral (VL), mucocutaneous (MCL), and cutaneous leishmaniasis (CL) were taken from different localities within the endemic areas. (A,B) [66] Ascites and enlarged reticuloendothelial organs (particularly spleen and liver), (C) [67] post-Kala-azar dermal leishmaniasis, (D) [67] an MCL mutilating lesion, (E) a nonhealing CL lesion, (F,G) lupoid leishmaniasis (leishmaniasis recidivans), and (H) an ulcerative cutaneous lesion.

*Leishmania* misdiagnosis can occur due to various factors, such as overlapping symptoms with other diseases, lack of awareness among healthcare providers, variability in clinical presentation, and limitations in diagnostic tools. Here are some key points on *Leishmania* misdiagnosis: (i) Clinical symptoms: Leishmaniasis can present with diverse clinical manifestations, including skin ulcers, fever, weight loss, hepatomegaly, splenomegaly, and anemia. These symptoms may mimic other diseases like malaria, tuberculosis, or bacterial infections, leading to misdiagnosis. (ii) Limited diagnostic tests: Diagnosis of leishmaniasis can be challenging due to the lack of sensitive and specific diagnostic tools in endemic regions. (iii) Misdiagnosis can occur when relying solely on clinical symptoms without intrinsic confirmatory tests like serology, molecular, or biochemical techniques. (iv) Geographic variation: *Leishmania* species have different geographic distributions and clinical forms, which can lead to misclassification [62]. (v) Diagnostic challenges: The limited availability of sensitive and specific diagnostic tools in endemic areas makes diagnosing leishmaniasis difficult. Depending solely on clinical symptoms without confirmatory intrinsic tests may lead to misdiagnosis [65]. Leishmaniasis, acute or chronic, mimics a variety of disease conditions (Figure 3) [35].

### 5.2. Natural Events

#### 5.2.1. Climate Change and Drought

Drought, earthquakes, and climate change can all have an impact on the spread of leishmaniasis. The natural equilibrium and human settlements may be upset by these calamities, which may alter vector habitats and human behavior and raise the possibility of leishmaniasis transmission. For instance, population displacement caused by earthquakes may result in overcrowding in makeshift shelters with subpar sanitation, which may encourage the spread of the illness. Climate change has the potential to modify temperature and precipitation patterns, which in turn can impact the distribution and quantity of sandfly vectors.

The ZCL caused by *L*. major transmission can be greatly impacted by drought and the subsequent community abandonment. Reduced vegetation and water supplies are two environmental changes brought on by drought that may affect the sandfly habitats, which are the vectors that spread the parasite. The dynamics of interactions between humans and sandflies may be impacted by these modifications, which might raise the risk of ZCL transmission [68]. Overall, the complicated interaction between environmental variables, human behavior, and disease transmission dynamics is highlighted by the possibility that village abandonment and drought-induced environmental changes might facilitate the spread of ZCL [69].

The phenomenon of drought causing the abandonment of villages can have complex effects on the prevalence of different *Leishmania* species. The abandonment of villages may lead to the elimination of *Leishmania major*, a species that typically thrives in arid regions. However, the spread of risk for *L. tropica*, which is known to be prevalent in more urban or peri-urban areas, may increase as people migrate to other regions in search of resources or livelihood opportunities. This shift in human population dynamics can potentially alter the distribution and prevalence of different *Leishmania* species in the affected areas [6]. Some strategies to reduce the risk of migration among villagers include: (i) Economic development: Implementing programs that promote economic development in rural areas can create job opportunities and improve living conditions, reducing the need for migration. (ii) Infrastructure development: investing in infrastructure such as roads, schools, healthcare facilities, and access to clean water can enhance the quality of life in rural areas and discourage migration. (iii) Agricultural support: Providing support to farmers through training, access to resources, and technology can increase agricultural productivity and income, making it more sustainable for villagers to stay in their communities. (iv) Education and skill development: Offering education and skill development programs can empower villagers with the necessary tools to find employment locally and contribute to their community's growth. (v) Social support systems: Establishing social support systems, such as community programs, healthcare services, and social safety nets, can help villagers address challenges and improve their overall well-being [70, 71].

#### 5.2.2. Earthquakes

Following an earthquake, there can be an increased risk of outbreaks of leishmaniasis in affected areas. The results offered in the present study strongly recommend that, after the earthquake, significant health risks emerged in the Bam [72, 73] and Abdanan [74] districts and nearby areas [75, 76], leading to outbreaks of CL. These contributing factors included favorable environmental conditions, poor hygiene practices, changes in individual behavior, and the influx of nonimmune populations. These factors have the potential to trigger outbreaks in existing areas and establish new areas of concern. The epidemiological and clinical characteristics of the disease, including gender, age, location, severity, and duration of lesions, underwent notable changes postearthquake [77]. It is crucial to implement thorough surveillance, early diagnosis, and timely treatment in high-risk areas to prevent and manage outbreaks of CL, especially in endemic regions following a major earthquake. Similarly, the incidence of infectious diseases, including VL, gradually or abruptly amplified following earthquakes [31, 78].

#### 5.2.3. Deforestation

**Figure 4:**
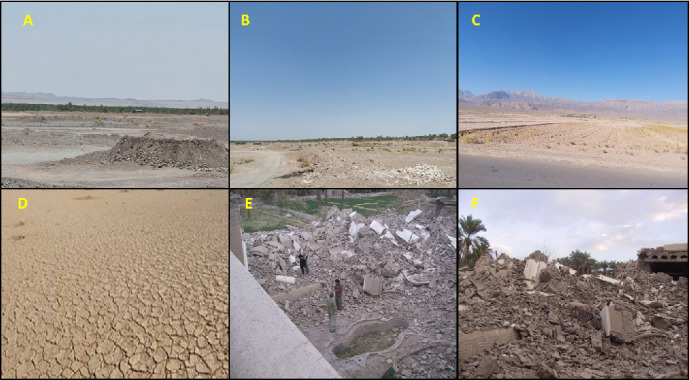
(A,B) Natural and man-made events induced: deforestation, (C,D) desertification, (E,F) earthquakes provide suitable conditions for resting and breeding sandfly vector transmissions of different forms of leishmaniasis.

Deforestation refers to the process by which vegetation in drylands, that is, arid and semi-arid lands, such as grasslands or shrublands, decreases and eventually disappears and has been linked to an increased risk of leishmaniasis transmission. The removal of vegetation and forests can disturb the natural habitats of sandflies, the vectors responsible for transmitting the *Leishmania* parasite to animals and/or humans (Figure 4) [79]. Deforestation can lead to changes in the ecological balance, creating new breeding grounds for sandflies and augmenting human exposure to the disease. Furthermore, deforestation can relocate wildlife, potentially bringing infected animals closer to human populations. These factors contribute to the higher incidence of leishmaniasis in areas affected by deforestation [80]. It is important to consider the environmental impact of deforestation in the context of disease transmission and implement measures to mitigate the risk of leishmaniasis in deforested regions. It is essential to address the root causes of deforestation and implement sustainable land management practices to preserve natural habitats and reduce the proliferation of sandfly vectors. In addition, community-based interventions focusing on vector control, health education, improved housing, early diagnosis, and treatment can effectively reduce the risk of leishmaniasis transmission in areas affected by deforestation. It could be an economic process where local populations and public health authorities may have limited control.

## 6. Impact Events

### 6.1. Immune System Deficiency

In the context of leishmaniasis transmission, the immune system plays a critical role in determining the infection's outcome. The host's immune response is key in controlling the disease's spread. Individuals with a robust and efficient immune response are better equipped to combat *Leishmania* parasites, reducing the chances of disease transmission. Immunity to leishmaniasis is predominantly mediated by cell-mediated immune responses, involving T cells, macrophages, and cytokines [81, 82]. These immune cells collaborate to identify and eliminate the parasites, halting their replication and spread within the host. A strong immune response can result in infection resolution and the establishment of lasting immunity against future encounters with the parasite [83]. Conversely, individuals with compromised immune systems, such as those with HIV/AIDS or other immunosuppressive conditions, are more vulnerable to severe forms of leishmaniasis and may act as reservoirs for ongoing disease transmission [84]. In such instances, the absence of an effective immune response allows the parasites to multiply unchecked, heightening the risk of transmission to others.

Hence, the interaction between immunity and the acquisition of leishmaniasis transmission is essential for comprehending the disease's dynamics. A robust immune response not only defends the host against infection but also significantly restricts the dissemination of leishmaniasis among populations [81]. Initiatives aimed at boosting immunity through vaccination or alternative immunomodulatory approaches can aid in managing and preventing the transmission of leishmaniasis [85].

In the framework of the immune response, the age pyramid and the decline in leishmaniasis transmission play a complicated interplay impacted by several variables. Due to variations in immunological responses between age groups, the age pyramid, which depicts the distribution of age groups in a community, can generally influence the spread of leishmaniasis. Due to their weakened immune systems, young children and the elderly are frequently more vulnerable to severe types of leishmaniasis [86]. On the other hand, healthy individuals could have more robust immune responses [81], which could aid in managing the illness and lowering the incidence of transmission. Improved access to healthcare, vaccine campaigns, vector control strategies, and herd immunity can all have an impact on the contraction of leishmaniasis transmission [87].

The cooccurrence and worsening of leishmaniasis alongside other infectious and non-infectious diseases can significantly impact patient well-being. When individuals are already immunocompromised, such as in cases of conditions like HIV/AIDS, the presence of leishmaniasis can result in more severe symptoms and complications. Moreover, coinfection with additional infectious diseases such as malaria, tuberculosis, substance abuse, and diabetes can further complicate the treatment and care of leishmaniasis. Healthcare providers must conduct thorough assessments and monitoring of patients with coinfections to ensure the implementation of suitable treatment strategies [84, 88–91].

To address the issue of coinfection and exacerbation of leishmaniasis with other infectious and non-infectious diseases, various steps can be taken: (i) Prevention measures: Implementing strategies to prevent the spread of leishmaniasis, such as vector control and personal protective measures, can help lower the disease's prevalence. Also, promoting overall health and hygiene practices can aid in preventing coinfections with other diseases. (ii) Early identification and diagnosis: Timely recognition and diagnosis of leishmaniasis and any coinfections are essential for initiating appropriate treatment. Healthcare professionals should be trained to identify the symptoms of these diseases and conduct thorough assessments to detect co-infections. (iii) Holistic treatment approach: Developing treatment plans that address both leishmaniasis and any coexisting infections is crucial for enhancing patient outcomes. Collaboration among specialists in various fields, including infectious diseases, immunology, and primary care, can ensure a comprehensive and coordinated treatment strategy. (iv) Research and education: Continued research into the interactions between leishmaniasis and other diseases is necessary to deepen our understanding of coinfections and develop more effective treatment approaches. Educating healthcare providers and the public about the risks of coinfections and the importance of early detection and treatment is also vital [22, 39, 55, 91–97].

### 6.2. Parasite Drug Resistance

Drug resistance in *Leishmania* species can arise from inappropriate or insufficient use of antileishmanial medications, resulting in the survival and proliferation of resistant strains [98]. The emergence of drug-resistant *Leishmania* strains can complicate treatment efforts and aid in the disease's spread. Furthermore, drug-resistant strains can persist in the environment and contribute to the continuous transmission of leishmaniasis [99].

Collaborative initiatives involving healthcare professionals, researchers, and public health agencies play a vital role in addressing drug-resistant *Leishmania* strains and managing the spread of leishmaniasis.

The implementation of strategies, such as drug resistance pattern surveillance, the development of novel antileishmanial drugs, and strict adherence to treatment protocols, is crucial in addressing the problem of drug resistance in leishmaniasis [100]. These measures are intended to minimize the emergence and spread of resistant strains. Working together, healthcare professionals, researchers, and public health authorities can effectively battle drug-resistant types of leishmaniasis and manage the disease's spread.

To address the problem of drug resistance in leishmaniasis, strategies such as tracking drug resistance trends, developing new antileishmanial drugs, and adhering to treatment protocols to prevent the emergence and spread of resistant strains are essential. To address drug-resistant *Leishmania* strains and other health issues, collaborative actions including healthcare providers, researchers, and public health organizations are essential [98, 101].

Parasite drug resistance often occurs in anthroponotic leishmaniasis compared to zoonotic forms.

In anthroponotic leishmaniasis, where the disease is primarily transmitted between humans, there is a higher likelihood of parasite resistance developing compared to zoonotic types, where the disease is transmitted between animals and humans. This is because, in anthroponotic transmission, the parasites are constantly exposed to human immune responses, and parasites in nonhuman vertebrates also face immune pressures, though potentially different in nature and intensity. It is important to implement different strategies for the control of zoonotic leishmaniasis versus anthroponotic forms of leishmaniasis [102].

When it comes to controlling zoonotic leishmaniasis, which is transmitted between animals and humans, strategies often focus on targeting the animal reservoirs of the disease [19], such as implementing measures to control the population of infected animals or using insecticide to reduce the vector population. On the other hand, for anthroponotic leishmaniasis, which is primarily transmitted between humans, strategies may involve improving sanitation and hygiene practices, implementing vector control measures, and providing access to effective treatment for infected individuals. Tailoring control strategies to the specific transmission dynamics of each form of leishmaniasis is crucial in effectively managing and preventing the spread of the disease [103].

To diminish the risk of drug resistance in leishmaniasis, the following strategies can be implemented: (i) Proper diagnosis and treatment: Ensure accurate diagnosis of leishmaniasis and prescribe appropriate treatment regimens based on the species of the parasite and the patient's condition. Avoid overuse or misuse of antileishmanial drugs. (ii) Combination therapy: Use combination therapy with multiple drugs that have different mechanisms of action to reduce the likelihood of resistance development. (iii) Monitoring and surveillance: Regularly monitor treatment outcomes and conduct surveillance for drug resistance to detect any emerging resistance patterns early on. (iv) Research and development: Invest in research and development of new antileishmanial drugs with novel mechanisms of action to combat drug-resistant strains. (v) Education and awareness: educate healthcare providers, patients, and communities about the importance of adherence to treatment regimens and the risks associated with drug resistance. (vi) Drug resistance testing: Conducting drug sensitivity testing to identify the specific drugs to which the parasite causing leishmaniasis is resistant can help guide treatment decisions. This information can help healthcare providers select alternative medications that are more likely to be effective [104].

Nonadherence or poor treatment adherence is a significant factor in effectively treating leishmaniasis. Patients must follow their prescribed treatment regimen consistently to ensure successful outcomes and reduce the risk of disease recurrence. Poor treatment adherence refers to when patients do not entirely follow their prescribed treatment plan as instructed by healthcare providers. This can involve missing doses, not completing the full course of medication, taking medications at incorrect times, or stopping treatment prematurely. In the context of leishmaniasis, poor treatment adherence can lead to treatment failure, increased risk of drug resistance, and disease relapse [38].

Increasing adherence to treatment in patients infected with leishmaniasis, especially when the treatment is challenging or perceived as poor, requires a multifaceted approach. Here are some strategies that can help improve adherence: (i) Patient education: Providing clear and comprehensive information to patients about the importance of adhering to the treatment regimen, the potential consequences of non-adherence, and the expected benefits of completing the treatment can help motivate patients to follow through with their medication. (ii) Simplifying the treatment regimen: If possible, simplifying the treatment regimen by reducing the number of medications, dosing frequency, or duration of treatment can make it easier for patients to adhere to the prescribed regimen. (iii) Support and counseling: Offering emotional support, counseling, and regular follow-up appointments can help patients feel interested and supported throughout their treatment journey. (iv) Addressing barriers: Identifying and addressing any barriers that may be preventing patients from adhering to their treatment, such as financial constraints, transportation issues, or side effects of the medication, can help improve adherence. Healthcare providers can use tools such as medication reminders, pill organizers, or mobile health applications to help patients stay on track with their treatment [39, 105].

Caring for immunocompromised patients against leishmaniasis requires a comprehensive approach to minimize the risk of infection and manage the disease effectively. Here are some key strategies to consider: (i) Preventive measures: Immunodeficient patients are more susceptible to infections, including leishmaniasis. It is important to educate patients about the risks of contracting the disease and advise them on preventive measures such as avoiding sandfly bites by using insect repellent, wearing long sleeves and pants, and staying indoors during peak biting times. (ii) Regular screening: Immune-deficient patients should undergo regular screening for leishmaniasis to detect any signs of infection early. This can help in prompt diagnosis and treatment, reducing the risk of complications. (iii) Immunomodulatory therapy: Depending on the underlying cause of immune deficiency, patients may benefit from immunomodulatory therapy to boost their immune response against leishmaniasis. This can include medications or treatments that help strengthen the immune system's ability to fight off infections. (iv) Prompt treatment: If an immunocompromised patient is diagnosed with leishmaniasis, it is crucial to start treatment promptly to prevent the infection from spreading and causing severe complications. Healthcare providers may recommend specific medications or treatment regimens tailored to the patient's condition. (v) Close monitoring: Immune-deficient patients with leishmaniasis should be closely monitored throughout their treatment to ensure that the infection is responding to therapy and to manage any potential side effects or complications. Regular follow-up appointments and laboratory tests may be necessary to track progress. (vi) Supportive care: Providing immune-deficient patients with adequate nutritional support, hydration, and rest can help support their overall health and immune function, making them better equipped to fight off infections like leishmaniasis [104, 106].

### 6.3. Insecticide-Resistant Sandflies

Controlling leishmaniasis is extremely difficult due to the disease's transmission and spread by sandflies that are resistant to insecticides. Sandfly insecticide resistance can impede efforts to reduce vectors, resulting in higher rates of transmission and a wider geographic distribution of leishmaniasis [107]. To solve this problem and stop the illness from spreading further, tactics including integrated vector control, the use of substitute pesticides, and the monitoring of insecticide resistance are essential. Developing successful control methods against leishmaniasis spread by insecticide-resistant sandflies requires cooperation between public health officials, researchers, and communities [108–110].

### 6.4. Various Clinical Forms

Through the bite of an infected sandfly, people can get leishmaniasis. Environmental variables, host vulnerability, and the existence of aberrant clinical manifestations of the illness can all have an impact on the transmission and spread of leishmaniasis. MCL due to *L. braziliensis* by contiguity or metastasis is a typical tissue tropism. The parasite that causes MCL can spread to mucous membranes, which can result in deformities and other consequences. There is a higher chance of disease transmission with this version of the illness, as it can be more difficult to identify and cure. Internal organs, including the spleen, liver, lymph nodes, and bone marrow, are impacted by VL, commonly referred to as Kala-azar. These are also typical tropisms of *L. donovani* and *L. infantum*. This severe type of illness can impair immunity, increasing a person's vulnerability to subsequent illnesses and parasite spread. The existence of atypical clinical variants of leishmaniasis might provide challenges to the management and control of the illness, as they could necessitate specific medical attention and monitoring protocols. It is crucial to comprehend the dynamics of leishmaniasis transmission and dissemination in the context of typical clinical manifestations to put preventative measures into practice and enhance patient outcomes [65, 111, 112].

Leishmaniasis transmission and spread in the context of post-Kala-azar dermal leishmaniasis (PKDL) and lupoid leishmaniasis can vary depending on the specific type of leishmaniasis and the geographical region. Both forms are highly resistant to treatment, and humans act as principal hosts in the spread of the disease. After VL (Kala-azar) is successfully treated, a complication known as PKDL may develop [113]. It is distinguished by skin lesions that may serve as a parasite reservoir, perhaps resulting in sandfly transmission and further disease dissemination. Like other types of leishmaniasis, PKDL can be spread by the bite of an infected sandfly. Conversely, lupoid leishmaniasis is an uncommon kind of CL caused by *L. tropica* that is distinguished by persistent, noduloid skin lesions. The primary mode of transmission and dissemination of organism is also through the biting of infected sandflies, which act as parasite vectors [114]. Preventive measures such as vector control, early diagnosis, and treatment of leishmaniasis cases are essential in controlling the transmission and spread of the disease, including in the framework of PKDL and lupoid leishmaniasis [39, 115]. It is important to consult healthcare professionals and public health authorities for specific guidance on prevention and management strategies for leishmaniasis in different contexts.

### 6.5. Lack of Micronutrients

Inadequate levels of essential nutrients like iron, zinc, and selenium can influence the body's immune response to *Leishmania* infection. These nutrients are vital for key immune functions, such as generating reactive oxygen species, managing immune cell behavior, and preserving cell structure. A shortage of these crucial elements may compromise the immune system's capacity to fight *Leishmania* parasites, heightening the risk of infection and potentially worsening disease severity. Thus, it is crucial to maintain sufficient levels of these essential nutrients to support a strong immune defense against *Leishmania*. Various factors like co-infections, malnutrition, immunosuppression, and environmental conditions can worsen leishmaniasis. Malnutrition, especially deficiencies in essential nutrients, can hinder immune function, reducing the body's ability to combat the parasite. Immunosuppression from underlying health issues or medications can heighten susceptibility to severe forms of leishmaniasis. These combined factors can exacerbate leishmaniasis outcomes, making the disease harder to manage and treat.

### 6.6. Asymptomatic Carriers

The role of asymptomatic carriers in the transmission of leishmaniasis is not well understood. It has been observed that a significant number of individuals in endemic areas are infected with *Leishmania* but do not exhibit any symptoms of CL or VL. Asymptomatic carriers of CL and VL refer to ACL and AVL, although asymptomatic dogs in urban America, VL is also an actual issue [7, 116]. The ratio of asymptomatic infections to clinical cases of VL varies widely, ranging from 4:1 in Kenya to 50:1 in Spain. In certain studies, parasite presence was detected in a small percentage of seropositive carriers through PCR testing, such as 2.5% in Bihar and 12.5% in Iran. An important question arises regarding whether asymptomatic carriers can transmit the infection to sand flies and, if so, what control measures should be implemented. Controlling asymptomatic carriers of leishmaniasis involves several key measures, including: (i) Surveillance and screening. (ii) Treatment. (iii) Education and awareness. (iv) Vector control. (v) Follow-up and monitoring [117, 118].

## 7. Brief Discussion

**Figure 5:**
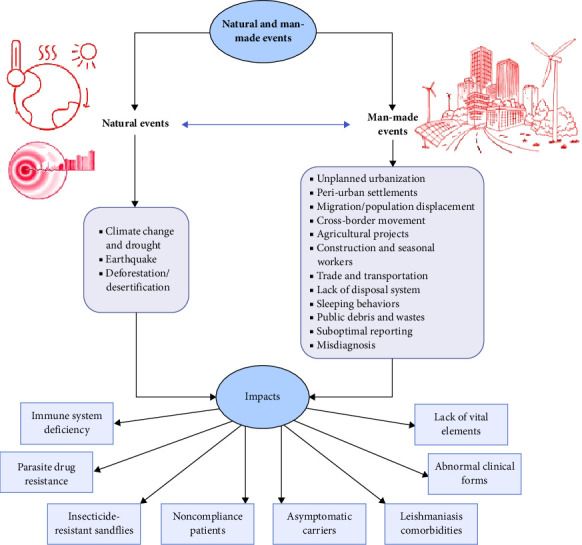
Schematic drawings of natural and man-made events, associated precipitating factors, and consequent impacts.

Both natural and man-made events can have significant impacts on the transmission and incidence of leishmaniasis. Changes in temperature and precipitation patterns can influence the distribution and abundance of sandfly vectors, affecting leishmaniasis transmission. Warmer temperatures can expand the geographic range of sandflies, while increased rainfall can create breeding sites for them. Deforestation can alter the natural habitats of sandflies and their animal reservoirs, pushing them closer to human settlements and increasing the risk of disease spread. Events like floods, hurricanes, droughts, and earthquakes can disrupt ecosystems, leading to population displacements, crowded living conditions, and poor sanitation, all of which can enhance the spread of leishmaniasis. Urbanization: Rapid urbanization can create favorable conditions for sandfly breeding, as unplanned settlements often lack proper waste management and sanitation facilities, increasing the risk of disease transmission. Civil unrest, conflict, and refugee crises can lead to overcrowded living conditions, poor access to healthcare, and disruption of disease control programs, exacerbating the spread of leishmaniasis. Changes in agricultural practices, such as irrigation projects or land-use changes, can create new habitats for sandfly vectors, increasing the risk of leishmaniasis dispersion among agricultural workers and nearby communities. Inherent and manufactured events play crucial roles in shaping the epidemiology of leishmaniasis (Figure 5). Understanding these impacts is essential for effective disease control and prevention strategies. Mitigating the effects of climate change, promoting sustainable land-use practices, improving living conditions in urban and rural areas, strengthening healthcare systems in conflict-affected regions, and enhancing surveillance and control programs are key steps in addressing the complex interplay between natural and man-made events on leishmaniasis transmission [7].

## 8. Conclusion

In conclusion, leishmaniasis is closely associated with both human activities and natural events, positioning it as a major social and public health issue affecting a large population in tropical countries. Significant environmental alterations, whether caused by humans or nature, can interact to trigger leishmaniasis, leading to its emergence and re-emergence in new areas. Consequently, enhancing public health awareness, particularly in high-risk regions, along with implementing active and passive surveillance, ensuring early diagnosis, and providing timely and effective treatment for patients, are vital measures for controlling the disease.

## Data Availability

All data generated through the review process is presented within the manuscript.
